# Dietary Behavior of Adolescents in the Qassim Region, Saudi Arabia: A Comparison between Cities with and without the Healthy Cities Program

**DOI:** 10.3390/ijerph18189508

**Published:** 2021-09-09

**Authors:** Ibrahim Alasqah, Ilias Mahmud, Leah East, Nada Alqarawi, Kim Usher

**Affiliations:** 1 Department of Public Health, College of Public Health and Health Informatics, Qassim University, Al Bukairiyah 52741, Saudi Arabia; i.emdadulhaque@qu.edu.sa; 2 School of Health, University of New England, Armidale, NSW 2351, Australia; leah.east@une.edu.au (L.E.); kusher@une.edu.au (K.U.); 3 Department of Basic Medical Sciences, Unaizah College of Medicine and Medical Sciences, Qassim University, Unaizah 56432, Saudi Arabia; n.alqarawi@qu.edu.sa

**Keywords:** Healthy Cities Program, adolescents, dietary behavior, nutrition, Saudi Arabia

## Abstract

This study reports dietary behaviors of adolescents in the Qassim region, Saudi Arabia, and comparison of these behaviors between cities with and without the Healthy Cities Program (HCP). We surveyed 1133 school-attending adolescents aged 15–19, using a multi-staged cluster sampling with probability proportionate to size. Prevalence of daily breakfast consumption was 27.7% among the adolescents. Prevalence of daily vegetables, fruits and milk or milk products consumption was 35.9%, 28.6% and 51.1%, respectively. Meanwhile, the prevalence of daily consumption of fast-food and carbonated drinks was 7.5% and 37.1%, respectively. There was no significant association between living in the healthy cities and daily intake of breakfast (OR: 1.15, 95% CI: 0.87–1.53), fruits (OR: 1.02; 95% CI: 0.77–1.36), vegetables (OR: 1.27; 95% CI: 0.98–1.67), or milk/milk products (OR: 1.0; 95% CI: 0.77–1.29); and the daily intake of fast-food (OR: 0.81; 95% CI: 0.49–1.35) or carbonated drinks (OR: 0.80; 95% CI: 0.60–1.05). These findings warrant further in-depth evaluation of the HCP in the Qassim region of Saudi Arabia.

## 1. Introduction

Adolescents experience various biological, psychosocial and cognitive changes that determine their nutritional needs and can also influence their dietary behaviors [1]. Many unhealthy dietary behaviors are prevalent among adolescents, including skipping meals; snacking; eating out; lower intake level of fruits, vegetables and whole grains; and having higher intake levels of energy-dense, unhealthy foods and beverages [2]. Healthy dietary behaviors include eating a variety of foods from the five food groups in an appropriate amounts and frequencies. The insufficient intake of fruit, vegetables, legumes, nuts and grains and the consumption of foods that contain high amounts of salt, sugar and fats are considered unhealthy dietary behaviors [3]. An unhealthy diet is one of the major risk factors for numerous non-communicable diseases (NCDs), including cardiovascular diseases, cancer and diabetes, and is the leading cause of obesity [4]. Adolescents who engage in unhealthy dietary behaviors are at risk of developing health issues, including growth and puberty delays, anemia and obesity [5,6]. Various risky behaviors start during adolescence and may become lifelong habits, increasing the risk of morbidity and mortality [7,8]. Several studies have shown a link between body weight and eating habits [9]. Additionally, socio-economic and cultural factors influence the development of eating habits [10]. The regular consumption of fruits and vegetables by children and adolescents has been associated with a lower risk of NCDs in later stages of life [11]. A recent study among adolescent students indicated that a high proportion of adolescents did not meet the recommended daily intake levels for vegetables and fruits [12]. Evidence shows that regular breakfast consumption by adolescents, compared with skipping breakfast, minimizes their risk of being overweight [13,14]. Despite its significance to their health and wellbeing, adolescents tend to skip breakfast more than any other meal [15]. Studies also report that most adolescents in Saudi Arabia do not adhere to the recommendations for healthy dietary behaviors [16,17].

The World Health Organization (WHO) introduced the Healthy Cities Program (HCP) in 1986 as an effective method of enhancing health equity in urban areas. The HCP plays an important role in developing and maintaining political, professional, and technical partnerships to accomplish goals set to promote health. In addition, the program helps with the development of a supportive community in which innovative motives for developing community infrastructure are accompanied by the implementation of comprehensive and inclusive methods. This program requires thorough and effective systematic efforts to address health inequalities, with a focus on urban poverty and the needs of vulnerable groups. The HCP also highlights the key effects of poor health from socio-economic and environmental perspectives and puts health at the center of the agenda for growth in the economy and urbanization. To qualify as a healthy city, a city must complete three steps. The process begins by joining the regional health and urbanization movement and the second step is to join the regional healthy city network. Finally, the city is evaluated to determine whether it can be considered a healthy city and to be recognized by the WHO regional office [18]. The HCP was launched in two Saudi Arabian cities in 1998 and has expanded the coverage across the country, with 32 cities currently operating the HCP [19,20]. Nine of these cities have recently been certified by the WHO as healthy cities after passing the 80 points criteria set by the WHO. These criteria include health promotion activities, including school-based health promotion, targeting dietary behaviors (Sarah I. Alkhoreyaf, Assistant Supervisor of Healthy Cities Program, e-mail communication, 23 December 2020) [19].

To date, no research has been published which evaluated the impact of the HCP on adolescents’ health, particularly their dietary behaviors, in the Kingdom of Saudi Arabia (KSA). The aim of this article was to investigate the dietary behaviors of adolescents in the Qassim region of the KSA and compare these behaviors between the cities with and without the HCP. This article also aims to examine the socio-demographic predictors of different dietary behaviors among adolescents in the Qassim region, KSA.

## 2. Materials and Methods

### 2.1. Setting

This study took place in six cities within the Qassim region, KSA. The Qassim region is in the center of the country, with an estimated population of 1.42 million living in cities and other rural communities [21]. Approximately one-quarter of the population is aged between 10 and 24 years. Data were gathered over six months, from April 2017 to September 2017.

### 2.2. Study Design and Sampling

**Figure 1:**
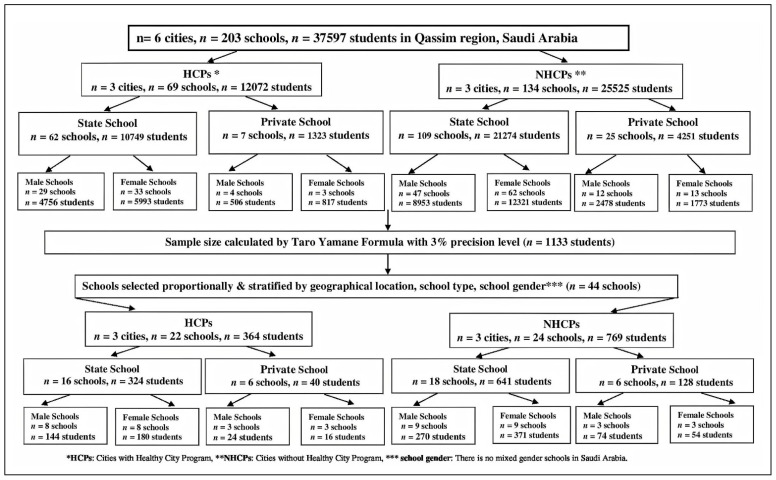
The sampling frame and sampling.

This study is part of a larger cross-sectional survey to assess adolescent students’ health risk behaviors, specifically smoking, diet and physical inactivity, in the Qassim region, KSA [22,23]. We applied multi-stage cluster sampling to recruit adolescents enrolled in high schools in six cities within the Qassim region. Albukayriah, Onaiza and Almedhnab were implementing the HCP, while Buraidah, Alrass and Albadea were not implementing the HCP. The total population of high schools’ students in the six selected cities in Qassim region was 37,597 at the time of conducting this study. Most of the study population was living in cities without the HCP (68%) while 32% were living in the cities with the HCP. The sample size was calculated with the Taro Yamane formula [24]. The minimum sample size required for a probability value of 0.05 was 396 and that needed for a probability value of 0.03 was 1079. Because a considerably larger data set provides more accurate prevalence estimates and greater power in the subsequent multivariable statistical analysis, we targeted 1133 students anticipating a 5% non-response rate. To obtain a representative sample of students, we used a stratified sampling technique to select schools and participants based on their geographic locations within each participated city and the type of the school (state vs. private) and whether it is a male or female school, since there are currently no co-educational schools in the KSA. We selected students randomly, based on a probability that was proportionate to the size of the chosen schools. Figure 1 presents a flow-chart of sampling. After obtaining consent from the students to participate in the study, a self-reported structured questionnaire (Supplementary Material S1) was administered to the participants.

### 2.3. The Instrument

The structured questionnaire covered aspects of dietary behaviors and explored breakfast consumption and food choices among the students. The questionnaire items assessed students’ daily consumption of breakfast and daily consumption of different types of food, such as fruits, vegetables, milk products, fast food and sugar sweetened carbonated/energy drinks. To assess these dietary behaviors, we asked the students to consider both in-school and out of school consumptions using the past seven days as reference period. Our questionnaire items were adapted from the Youth Risk Behaviors Survey developed by the CDC, USA [25], Health behavior in School-aged Children developed by the WHO [12] and the Global School-based Student Health Survey, which was developed by the WHO in collaboration with the CDC [26]. The initial questionnaire was pretested with 20 school going adolescents in two rounds. Pretesting was focused on examining whether the adolescents experience any difficulties in completing the questionnaire. After the first round of pretesting with 10 adolescents, the questionnaire was revised based on the feedback received from pretesting. Following minor adjustment in wording of the questions and response options, a second-round pretesting was done with another 10 adolescents to ensure that all problems identified in the previous round were rectified and no new problems were introduced.

### 2.4. Data Collection

Data were collected by distributing questionnaires to the randomly selected students at high schools in grades 10–12 (aged 15 to 19) in six cities in the Qassim region. The data for male students were collected by the first author, whereas a trained and experienced female research assistant gathered data from the female students. Detailed written instructions on how to complete the questionnaire were provided to the students. In addition, the instructions were given verbally to the students involved. The participating students recorded their responses without any assistance. No identifying information was gathered to ensure anonymity. Students who were not present during the data collection process and those under 15 years of age or over 19 years of age were excluded from the analysis.

### 2.5. Data Processing and Statistical Analysis

Data were processed using IBM SPSS Statistics for Windows, v.22.0 (IBM Corp., Armonk, NY, USA). Data were screened for missing values, outliers and collinearity. Participants’ socio-demographic and diet-related characteristics are presented as frequencies. The prevalence of different healthy and unhealthy dietary behaviors was reported with 95% confidence intervals (CIs). Multivariable logistic regression analyses were performed to investigate the predictors of daily breakfast consumption, predictors of healthy food intake, and predictors of energy-dense food intake among adolescents in Saudi Arabia. For multivariable logistic regression analyses, a *p* value of <0.05 were considered statistically significant. A comparison was made using the city classification (cities with and without the HCP) as a grouping variable. For the results of the regression analysis, odds ratios (ORs) with 95% Cis and p values were reported.

## 3. Results

### 3.1. Socio-Demographic Characteristics of the Adolescents

We surveyed 1133 adolescents attending school in six cities in the Qassim region of Saudi Arabia. They were aged between 15 and 19 years with a mean (±SD) age of 16.96 (±0.99) years. Half of these cities participate in the HCP. Less than one-third of our participants were living in the cities with the HCP. The majority of them were female (54.6%), were studying in state schools (85.0%), lived with both parents (86.8%), were Saudi nationals (91.4%) and had excellent academic performance (59.3%). Regarding their parents’ academic profile, 41.7% of the fathers and 38% of the mothers had completed a tertiary-level education (Table 1).

### 3.2. Predictors of Daily Breakfast Consumption among Adolescents

We found that slightly more than one-quarter (27.7%) of the adolescents ate their breakfast daily (Table 2). We performed multivariable logistic regression analyses to investigate the predictors of daily breakfast consumption among adolescents in the Qassim region, KSA (Table 3). We found no evidence of a significant association between living in cities with the HCP and the daily consumption of breakfast (OR: 1.15, 95% CI: 0.87–1.53) among the adolescents, after adjusting for the effect of other socio-demographic variables. In addition, there was no evidence of a significant association between daily breakfast consumption and the socio-demographic variables (city classification, age, nationality, school type, academic performance, or parental education) except sex. However, we found that females are less likely to consume breakfast daily (OR: 0.74; 95% CI: 0.57–0.97).

### 3.3. Predictors of Healthy Food Intake among the Adolescents

We found that the prevalence of the daily intake of vegetables, fruits and milk among adolescents was 35.9%, 28.6% and 51.1%, respectively. There was no evidence of a significant association between living in the cities with the HCP and the daily intake of fruits (OR: 1.02; 95% CI: 0.77–1.36), vegetables (OR: 1.27; 95% CI: 0.98–1.67), or milk/milk products (OR: 1.0; 95% CI: 0.77–1.29) after adjusting for the effect of other socio-demographic variables. However, we found that the likelihood of daily fruits intake decreased with increasing age (OR: 0.83; 95% CI: 0.73–0.96) after adjusting for the effect of all other socio-demographic variables. After adjusting for the confounding effect of other socio-demographic variables, we found that females (OR: 1.47; 95% CI: 1.14–1.90) and non-Saudi adolescents (OR: 1.90; 95% CI: 1.24–2.90) were more likely to eat vegetables daily. Regarding daily milk/milk product intake, we found significant associations with school type and maternal education level. The odds of daily milk or milk product intake among adolescents studying in private school was 1.46 (95% CI: 1.04–2.05) times that among adolescents studying in the state schools. The odds of daily milk or milk product intake among adolescents with mothers who had a tertiary-level education was 1.55 (95% CI: 1.01–2.36) times that among adolescents with mothers who had up to a primary level of education (Table 4).

### 3.4. Predictors of Energy-Dense Food Intake among Adolescents

The prevalence of daily fast-food intake and daily carbonated drink intake among adolescents were 7.5% and 37.1%, respectively. We found no evidence of a significant association between living in the cities with the HCP and daily fast-food (OR: 0.81; 95% CI: 0.49–1.35) or carbonated drink (OR: 0.80; 95% CI: 0.60–1.05) intake among the adolescents after adjusting for the effect of other socio-demographic variables. However, we found evidence of significant associations between daily fast-food intake and sex, academic performance, and nationality of the adolescents after adjusting for the effect of other socio-demographic variables. Females were 53% less likely to eat fast food daily than males (OR: 0.47; 95% CI: 0.29–0.75). Adolescents with excellent academic performance were 68% less likely to eat fast food daily than adolescents with average or poor academic performance (OR: 0.32; 95% CI: 0.12–0.85). Non-Saudi adolescents were 7% less likely to consume of fast food daily than Saudi adolescents (OR: 0.93; 95% CI: 0.86–0.93).

Regarding daily carbonated drink intake, we observed evidence of significant associations between the daily intake of carbonated drink and age, sex, academic performance and nationality of the adolescents after adjusting for the confounding effects of other socio-demographic variables. The likelihood of the daily intake of any carbonated drink increased with increasing age (OR: 1.15; 95% CI: 1.01–1.31). Female adolescents were 52% less likely to drink carbonated beverage daily (OR: 0.48; 95% CI: 0.37–0.63). Adolescents with excellent academic performance were 63% less likely to drink carbonated beverages daily (OR: 0.37; 95% CI: 0.18–0.75). Non-Saudi adolescents were 60% less likely to drink carbonated beverages daily (OR: 0.40; 95% CI: 0.24–0.68) (Table 5).

## 4. Discussion

Saudi society has experienced significant changes in all aspects of life, including dietary behaviors over the past decade. Several studies have explored this issue. However, none of these studies have compared adolescents’ dietary behaviors between cities with and without the HCP. This study investigated the dietary behaviors of Saudi adolescents living in the Qassim region in relation to the HCP. Our samples were randomly recruited, probability proportionate to the size, from the schools of three cities with the HCP and another three cities without the HCP in the Qassim region, Saudi Arabia.

We found that there was no evidence of statistically significant association between daily consumption of breakfast and living in cities with the HCP. Similarly, there was no significant association between living in cities with the HCP and the daily consumption of vegetables, fruits, or milk products. In addition, there was no statistically significant association between living in cities with the HCP and the daily intake of fast food or sugar sweetened carbonated drinks. The 80 points criteria that a city must fulfill to be recognized as a healthy city includes both community and school-based health promotion activities to promote healthy diet [27]. Therefore, it was expected that adolescents living in the cities with the HCP would show better dietary behaviors than those living in the cities without the HCP. However, our multivariable logistic regression analyses, after adjusting for the confounding effect of other socio-demographic variables, showed no significant differences between these two groups. This lack of differences could be due to the nationwide implementation of health promotion programs taking place irrespective of whether they are participating in HCP or not. This lack of effectiveness of the HCP could also be due to the challenges it faces. In the Eastern Mediterranean region, the HCP faces various challenges despite its widespread acceptance. These challenges include lack of institutionalization of the concepts and methodologies; lack of documentation and evidence building at the local level; and poor partnership with nongovernmental organizations, donors, UN agencies, academic and research institutions [28]. Nevertheless, our study findings warrant an in-depth evaluation of the process and outcomes of the HCP in the KSA.

Regular breakfast eating is linked to better health while skipping breakfast reduces concentration and school performance [29]. Breakfast was the most often missed meal among the participants in this study, with only (27.7%) adolescents eating breakfast daily. Similar findings were previously reported in various studies among Saudi adolescents. The prevalence of skipping breakfast among adolescents in Abha city was 49%, in Riyadh 47%, in Jeddah 54% and in Arar 40.9% [30,31,32,33]. The high prevalence of skipping breakfast among adolescents in Saudi Arabia could be due to the early start of the school day as students might not have enough time to have their breakfast before leaving home or not having the appetite. These barriers to breakfast intake are widely stated in the literature [29,34,35]. The findings from Arab countries are in line with our findings as more than 50% of adolescents in Bahrain [36], 62.5% in Qatar [34] and 61.72% in Lebanon [35] does not consume breakfast daily. Lower breakfast skipping rate (10–27%) was found among adolescents in United Arab Emirates and Jordan [37,38]. However, the differences in the prevalence rate between these studies could be due to the variation in skipping breakfast definition.

A large percentage of the Saudi population does not meet the recommended guidelines of vegetable and fruit intake. In our study, we noted that the prevalence of daily vegetable and fruit intake among the adolescents was 35.9% and 28.6%, respectively. A school-based survey study that was carried out in AlKhobar, Jeddah and Riyadh, the prevalence of daily vegetable intake was less than 25% in both boys and girls. The prevalence of daily fruit intake was 16% in males and 9.6% in females and this difference was statistically significant [17]. This study did not find any statistically significant association between gender and daily consumption of fruits. Our study also found that the likelihood of daily consumption of fruits declined with increasing age and this result is in line with findings from various studies [39,40,41]. A nationwide study in Saudi Arabia that interviewed 10,735 individuals aged 15 years and older found that only 5.2% and 7.5% consume the recommended amount of fruits and vegetables, respectively [42]. Findings from the Gulf countries are also in line with our findings: almost 75% of adolescents in Kuwait, United Arab Emirates and Qatar do not follow the recommended guidelines for daily consumption of vegetables and fruits [43]. Similar findings were also reported in seven African countries (Botswana, Kenya, Senegal, Swaziland, Tanzania, Uganda and Zambia) where 77.5% of adolescents did not consume the recommended daily amount of vegetables and fruits [44]. Our study findings suggest the urgent needs for a comprehensive program to improve the dietary practices among Saudi adolescents.

Dairy products provide our bodies with protein, fat, carbohydrates and phosphorus and are rich in various minerals including magnesium, potassium and zinc. In the present study, the prevalence of daily milk intake among adolescents was 51.1% and the same result was found among Saudi adolescents in Abha city [30]. Adolescents studying in private schools and those whose mothers had a higher level of education had higher odds of consuming milk/dairy products. Other studies among Saudi adolescents found that 58.4% in Riyadh and 59.25% in Dammam consumed milk/dairy products regularly [45,46]. In contrast, two studies conducted in Riyadh and Jeddah found that only 26.2% and 38.9% of the total sample drunk milk everyday [31,32]. In Bahrain and Qatar, the daily consumption of milk among adolescents was 33% and 24%, respectively [36,47]. 

The present study revealed that the prevalence of daily fast-food intake among adolescents was 7.5%. It is worth mentioning that females and those with excellent academic performance were less likely to eat fast-food daily. This is similar to the results reported by Al-Hazzaa et al. who reported that the prevalence of daily consumption of fast food in male and female adolescents was 9% and 6%, respectively. Furthermore, a study conducted in Al-Khobar, Jeddah and Riyadh across 2908 adolescents is also consistent with our findings which reported that the overall intake of fast food per week was significantly higher in males than in females [17]. The authors have attributed this difference to the fact that adolescent boys in Saudi Arabia have more opportunity than girls to go to fast food restaurants because of cultural norms [17]. In contrast, studies conducted among medical students in Damam and food science students in Riyadh reported that 91% and 66% of the participants were regularly eating fast food respectively [45,48]. A global study collected data from 180,164 adolescents in sixty-eight countries found that the overall prevalence of fast-food consumption was 50.1% [49]. The prevalence of consuming carbonated drinks in the present study is 37.1%. Female students and students with excellent academic performance are less likely to drink carbonated beverages daily. Other studies in Saudi Arabia revealed that a high percentage of adolescents regularly consume carbonated drinks, with 77.4% in Dammam, 89% in Riyadh [45,50]. The prevalence of sugar sweetened carbonated drink intake among adolescents in Oman, Qatar and the United Arab Emirates ranges between 50% and 65% [43]. The Global School-based Student Health Surveys in Africa, Asia, Oceania and Latin America between 2008 and 2015 shows that 42.8% drink carbonated drinks everyday [51]. A plausible reason for the high frequency of sugar sweetened carbonated drink consumption is that these drinks have been marketed as a main component of any meal and are handed out at the majority of events. However, these drinks contain considerable amounts of caffeine, artificial sweeteners, sugar, and amino acids [52]. These results suggest that there is a great need to enhance these dietary behaviors.

The strength of the present study is that it is the first study to compare the dietary behaviors between the adolescents living in the cities with and without the HCP. In addition, it included a relatively large sample with randomly selected participants. However, the study was limited by the inclusion of only adolescents from 15–19 years of age, which may mean that the sample is not representative of all adolescents. Another limitation is that it was conducted only in one region, which may increase the risk of bias. It is therefore important for future studies to target different regions and include a large sample of adolescents from all age groups.

## 5. Conclusions

We investigated the prevalence and determinants of different dietary behaviors of adolescents in the Qassim region, Saudi Arabia and compared these behaviors between adolescents living in the cities with and without the HCP. We found no evidence of a significant association between living in cities with the healthy city program and the adolescents’ daily consumption of breakfast, vegetables, fruits, milk, fast food and sugar sweetened carbonated drinks. The prevalence of the daily consumption of vegetables, fruit, and dairy products are low among the adolescents. In addition, there is a high prevalence of the consumption of sugar sweetened carbonated drinks. Although the HCP is defined by the process rather than any health and health behavior-related outcomes, the HCP is expected to improve these outcomes. Hence, it was expected that the adolescents living in the cities with the HCP to have better dietary behavior than the adolescents living in the cities with the HCP. Therefore, we recommend an in-depth evaluation of the HCP in Saudi Arabia, especially in regard to the outcomes for adolescents.

## Figures and Tables

**Table 1:** Socio-demographic characteristics of the adolescents.

Characteristics	Cities with HCP (*n* = 364) % (*n*)	Cities without HCP (*n* = 769) % (*n*)	Total (*n* = 1133) % (*n*)
Sex			
Female	53.8 (196)	55.0 (423)	54.6 (619)
Male	46.2 (168)	45.0 (346)	45.4 (514)
School type			
Private	11.5 (42)	16.6 (128)	15.0 (170)
State	88.5 (322)	83.4 (641)	85.0 (963)
Academic performance			
Average	3.6 (13)	3.0 (23)	3.2 (36)
Good	34.6 (126)	38.9 (299)	37.5 (425)
Excellent	61.8 (225)	58.1 (447)	59.3 (672)
Living with both parents			
Yes	87.9 (320)	86.2 (663)	86.8 (983)
No	12.1 (44)	13.8 (106)	13.2 (150)
Nationality			
Non-Saudi	6.6 (24)	9.5 (73)	8.6 (97)
Saudi	93.4 (340)	90.5 (696)	91.4 (1036)
Paternal education			
No formal education	17.6 (64)	16.4 (126)	16.8 (190)
Primary	4.7 (17)	7.5 (58)	6.6 (75)
Secondary	34.1 (124)	35.4 (272)	35.0 (396)
Tertiary	43.7 (159)	40.7 (313)	41.7 (472)
Maternal education			
No formal education	14.8 (54)	17.9 (138)	16.9 (192)
Primary	9.1 (33)	11.7 (90)	10.9 (123)
Secondary	31.9 (116)	35.4 (272)	34.2 (388)
Tertiary	44.2 (161)	35.0 (269)	38.0 (430)

**Table 2:** Dietary behaviors of adolescents in Qassim, Saudi Arabia.

Dietary Behaviors	Cities with HCP		Cities without HCP		Total	
	Prevalence (95% CI)	p *	Prevalence (95% CI)	p *	Prevalence (95% CI)	*p* *
Daily breakfast consumption	29.9% (25.3–34.9)	0.000	26.7% (23.6–29.9)	0.000	27.7% (25.1–30.4)	0.000
Daily vegetable intake	39.0% (34.0–44.2)	0.000	34.5% (31.1–37.9)	0.000	35.9% (33.1–38.8)	0.000
Daily fruit intake	28.3% (23.7–33.2)	0.000	28.7 (25.6–32.1)	0.000	28.6% (26.0–31.3)	0.000
Daily milk/milk product intake	51.4% (46.1–56.6)	0.637	51.0% (47.4–54.6)	0.614	51.1% (48.1–54.1)	0.476
Daily fast-food intake	6.6% (4.3–9.7)	0.000	7.9% (6.1–10.1)	0.000	7.5% (6.0–9.2)	0.000
Daily carbonated drink intake	33.8% (28.9–38.9)	0.000	38.6% (35.2–42.2)	0.000	37.1% (34.2–40.0)	0.000

* One-sample nonparametric tests (binomial).

**Table 3:** Predictors of daily consumption of breakfast among adolescents in Qassim, Saudi Arabia.

Predictors	Daily Consumption of Breakfast	
	OR (95% CI)	*p*-Value *
City classification		
Cities without HCP	1	
Cities with HCP	1.15 (0.87–1.53)	0.320
Age	0.95 (0.83–1.09)	0.495
Sex		
Male	1	
Female	0.74 (0.57–0.97) **	0.028
School type		
State	1	
Private	1.04 (0.72–1.50)	0.833
Academic performance		
Average	1	
Good	0.91 (0.42–1.98)	0.818
Excellent	0.98 (0.45–2.10)	0.950
Living with…		
Both parents	1	
One parent or other relatives	0.82 (0.54–1.24)	0.347
Nationality		
Saudi	1	
Non-Saudi	1.12 (0.71–1.77)	0.629
Paternal education		
No formal education	1	
Primary	0.66 (0.32–1.34)	0.248
Secondary	1.23 (0.78–1.94)	0.384
Tertiary	0.99 (0.62–1.58)	0.977
Maternal education		
No formal education	1	
Primary	0.86 (0.48–1.52)	0.594
Secondary	0.97 (0.61–1.54)	0.886
Tertiary	1.12 (0.70–1.77)	0.664

* Multivariable logistic regression analysis; ** Results are statistically significant at *p* < 0.05.

**Table 4:** Predictors of healthy food intake among adolescents in Qassim, Saudi Arabia.

Predictors	Daily Intake					
	Fruits		Vegetables		Milk/Milk Product	
	OR (95% CI)	*p*-Value *	OR (95% CI)	*p*-Value *	OR (95% CI)	*p*-Value *
City classification						
Cities without HCP	1		1		1	
Cities with HCP	1.02 (0.77–1.36)	0.874	1.27 (0.98–1.67)	0.073	1.0 (0.77–1.29)	0.997
Age	0.83 (0.73–0.96) **	0.010	1.01 (0.89–1.14)	0.904	0.97 (0.85–1.10)	0.564
Sex						
Male	1		1		1	
Female	1.22 (0.94–1.60)	0.139	1.47 (1.14–1.90) **	0.003	1.12 (0.88–1.43)	0.360
School type						
State	1		1		1	
Private	1.27 (0.89–1.81)	0.194	1.35 (0.96–1.90)	0.086	1.46 (1.04–2.05) **	0.028
Academic performance						
Average	1		1		1	
Good	0.92 (0.43–1.96)	0.833	0.95 (0.46–1.98)	0.899	1.06 (0.53–2.13)	0.864
Excellent	0.71 (0.34–1.50)	0.366	1.03 (0.50–2.13)	0.929	1.33 (0.67–2.65)	0.423
Living with…						
Both parents	1		1		1	
One parent or other relatives	1.05 (0.71–1.56)	0.804	1.07 (0.74–1.55)	0.705	1.37 (0.96–1.96)	0.086
Nationality						
Saudi	1		1		1	
Non-Saudi	1.43 (0.92–2.24)	0.110	1.90 (1.24–2.90) **	0.003	0.71 (0.46–1.09)	0.115
Paternal education						
No formal education	1		1		1	
Primary	0.82 (0.41–1.62)	0.562	0.96 (0.52–1.79)	0.902	0.73 (0.41–1.30)	0.286
Secondary	1.40 (0.88–2.21)	0.163	1.42 (0.92–2.20)	0.118	1.25 (0.83–1.89)	0.282
Tertiary	1.23 (0.77–1.97)	0.383	1.39 (0.89–2.15)	0.145	1.15 (0.76–1.75)	0.501
Maternal education						
No formal education	1		1		1	
Primary	1.05 (0.61–1.83)	0.857	0.71 (0.42–1.89)	0.190	1.60 (0.97–2.62)	0.065
Secondary	1.00 (0.63–1.60)	0.984	0.72 (0.47–1.10)	0.127	1.35 (0.89–2.03)	0.156
Tertiary	1.01 (0.63–1.62)	0.971	0.72 (0.46–1.12)	0.142	1.55 (1.01–2.36) **	0.043

* Multivariable logistic regression analysis; ** Results are statistically significant at *p* < 0.05 or *p* < 0.01.

**Table 5:** Predictors of energy-dense food intake among adolescents in Qassim, Saudi Arabia.

Predictors	Daily Intake			
	Fast Food		Carbonated Drink	
	OR (95% CI)	*p*-Value *	OR (95% CI)	*p*-Value *
City classification				
Cities without HCP	1		1	
Cities with HCP	0.81 (0.49–1.35)	0.425	0.80 (0.60–1.05)	0.104
Age	1.12 (0.89–1.41)	0.339	1.15 (1.01–1.31) **	0.032
Sex				
Male	1		1	
Female	0.47 (0.29–0.75) **	0.002	0.48 (.37–0.63) **	0.000
School type				
State	1		1	
Private	1.44 (0.82–2.53)	0.211	1.26 (0.88–1.78)	0.206
Academic performance				
Average	1		1	
Good	0.54 (.21–1.40)	0.202	0.65 (0.32–1.34)	0.244
Excellent	0.32 (.12–0.85) **	0.022	0.37 (0.18–0.75) **	0.006
Living with…				
Both parents	1		1	
One parent or other relatives	0.66 (0.31–1.43)	0.295	1.01 (0.70–1.45)	0.950
Nationality				
Saudi	1		1	
Non-Saudi	0.93 (0.86–0.93)	0.860	0.40 (0.24–0.68) **	0.001
Paternal education				
No formal education	1		1	
Primary	2.42 (0.87–6.75)	0.091	0.72 (0.39–1.33)	0.297
Secondary	1.30 (0.58–2.94)	0.528	1.04 (0.67–1.60)	0.867
Tertiary	1.19 (0.52–2.71)	0.685	1.01 (0.66–1.57)	0.945
Maternal education				
No formal education	1		1	
Primary	0.59 (0.21–1.71)	0.329	1.18 (0.71–1.97)	0.522
Secondary	1.20 (0.56–2.61)	0.632	0.82 (0.53–1.26)	0.370
Tertiary	1.26 (0.56–2.81)	0.576	0.75 (0.48–1.17)	0.207

* Multivariable logistic regression analysis; ** Results are statistically significant at *p* < 0.05 or *p* < 0.01.

## Data Availability

Data used in this study are available from the corresponding author on reasonable request.

## Supplementary Materials

The following are available online at https://www.mdpi.com/article/10.3390/ijerph18189508/s1, The questionnaire.
