# Supplementary material for: Dietary Behavior of Adolescents in the Qassim Region, Saudi Arabia: A Comparison between Cities with and without the Healthy Cities Program
Source: Int J Environ Res Public Health. 2021 Sep 9;18(18):9508. doi: 10.3390/ijerph18189508 (PMC8465802; doi:10.3390/ijerph18189508)
Supplement: Supplementary file 1 [file ijerph-18-09508-s001.zip › ijerph-1367022-supplementary.pdf]

International Journal of
Environmental Research and Public Health

MDPI

The Questionnaire

1. **What is your sex?**
    1) Male.
    2) Female.
2. **How old are you?**
    1) 15.
    2) 16.
    3) 17.
    4) 18.
    5) 19.
3. **In what class level of school are you?**
    1) Grade 10th .
    2) Grade 11th .
    3) Grade 12th.
4. **In what type of school are you?**
    1) Public school.
    2) Private school.
5. **What grade did you get in your recent academic evaluation?**
    1) Failure.
    2) Poor.
    3) Average.
    4) Good.
    5) Very good.
    6) Excellent.
    7) I do not know.
6. **In which city do you live in the Qassim?**
    1) Onaiza.
    2) Alrass.
    3) Buraidah.
    4) Albukayriah.
    5) Almedhnab.
    6) Albadea.
7. **With whom of your parents do you live?**
    1) Both mother and father.
    2) Father only.
    3) Mother only.

4) Neither of them.

8. What is your father's education level?

1) No formal education.
2) Primary education.
3) Lower secondary education.
4) Upper secondary education.
5) Bachelor degree or postgraduate degrees.

9. What is your mother's education level?

1) No formal education
2) Primary education.
3) Lower secondary education.
4) Upper secondary education.
5) Bachelor degree or postgraduate degrees.

10. What is your family's total monthly income?

1) <1333 USD.
2) 1333-2666 USD.
3) 2667- 3999 USD.
4) 4000-5333 USD.
5) >5333 USD.
6) I do not know.

11. What is your nationality?

1) Saudi.
2) Non-Saudi.

Dietary Behaviours

12. During the past 7 days, how often did you eat breakfast?

1) Never.
2) Rarely.
3) Some days.
4) Most days.
5) Everyday.

13. What is the main reason you do not eat breakfast?

1) I always eat breakfast.
2) I do not have time for breakfast.
3) I do not feel hungry.
4) There is not always prepared breakfast meal in my home.

14. How many days do you eat lunch during the week?

1) Never.
2) One day.
3) Two days.
4) Three days.
5) Four days.
6) Five days and more.

15. How many days do you eat dinner during the week?

1) Never.
2) One day.
3) Two days.
4) Three days.
5) Four days.
6) Five days and more

16. How many snacks (e.g. fatayer, sandwich, chips, etc.) do you usually have per day?

1) Never.
2) One.
3) Two.
4) Three.
5) Four.
6) More than four times.
7) I eat snacks, but not daily.

17. How many times do you eat fruits per day?

1) Never.
2) One.
3) Two.
4) Three.
5) Four.
6) More than four times.
7) I eat fruits, but not daily.

18. How many times do you eat vegetables (e.g. salad, vegetable stew, molokhiya, etc.) per day?

1) Never.
2) One.
3) Two.
4) Three.
5) Four.
6) More than four times.
7) I eat vegetables, but not daily.

19. During the past 30 days, did you eat less food, fewer calories, or foods low in fat to lose weight?

1) Yes.
2) No.

20. How many times do you drink sugar sweetened carbonated beverages (e.g. Pepsi, Coke, 7-Up, Miranda, etc.) per day during the past 7 days?

1) Never.
2) One.
3) Two.
4) Three.
5) Four.
6) More than four times.
7) I drink carbonated beverages, but not daily.

21. How many times do you drink energy drinks (e.g. Red Bull, Power Horse, etc.) per day?

1) Never.
2) One.
3) Two.
4) Three.
5) Four.
6) More than four times.
7) I drink energy drinks, but not daily.

22. How many times do you consume milk or milk products (e.g. yogurt, cheese, labnah, etc.) per day during the past 7 days?

1) Never.
2) One.
3) Two.
4) Three.
5) Four.
6) More than four times.
7) I consume milk products, but not daily.

23. During the past 7 days, on how many days did you eat fast food (e.g. burger, shawarma, pizza, etc.) from a fast food restaurant such as Mac, KFC, Herfy, Albaik etc.?

1) Never.
2) 1 day.
3) 2 days.
4) 3 days.
5) 4 days.
6) 5 days.

7) 6 days.
8) Daily.

24. During your school years, were you taught in any of your classes the benefits of healthy eating such as the benefits of eating fruits, vegetables and milk or milk products?

1) Yes.
2) No.

25. During your school years, were you taught in any of your classes the health risks of eating fast food from restaurants?

1) Yes.
2) No.

26. During your school years, were you taught in any of your classes the health risks of drinking carbonated beverages?

1) Yes.
2) No.

27. During your school years, were you taught in any of your classes the health risks of drinking energy drinks?

1) Yes.
2) No.

28. Do you think students need more health education regarding healthy diet?

1) Yes.
2) No.
